# Risk factors of anaemia and iron deficiency in Somali children and women: Findings from the 2019 Somalia Micronutrient Survey

**DOI:** 10.1111/mcn.13254

**Published:** 2021-08-17

**Authors:** James P. Wirth, Fatmata Sesay, Joshua Mbai, Sundus Ibrahim Ali, William E. S. Donkor, Bradley A. Woodruff, Zahra Pilane, Kheyriya Mohamed Mohamud, Ahmed Muse, Hamda Omar Yussuf, Warsame Said Mohamed, Rosmarie Veraguth, Serge Rezzi, Thomas N. Williams, Abdullahi Muse Mohamoud, Farhan Mohamed Mohamud, Melanie Galvin, Fabian Rohner, Yvonne Katambo, Nicolai Petry

**Affiliations:** ^1^ GroundWork Fläsch Switzerland; ^2^ UNICEF Mogadishu Somalia; ^3^ Brandpro Research Nairobi Kenya; ^4^ Ministry of Health Federal Government of Somalia Mogadishu Somalia; ^5^ Ministry of Health, Somaliland Hargeisa Somalia; ^6^ Ministry of Health, Puntland Garowe Somalia; ^7^ Swiss Vitamin Institute Epalinges Switzerland; ^8^ KEMRI‐Wellcome Trust Research Programme Kilifi Kenya

**Keywords:** anaemia, children, determinants, epidemiology, iron, micronutrients, risk factors, Somalia, women of childbearing age

## Abstract

There are limited data on the prevalence of anaemia and iron deficiency (ID) in Somalia. To address this data gap, Somalia's 2019 micronutrient survey assessed the prevalence of anaemia and ID in children (6–59 months) and non‐pregnant women of reproductive age (15–49 years). The survey also collected data on vitamin A deficiency, inflammation, malaria and other potential risk factors for anaemia and ID. Multivariable Poisson regressions models were used to identify the risk factors for anaemia and ID in children and women. Among children, the prevalence of anaemia and ID were 43.4% and 47.2%, respectively. Approximately 36% and 6% of anaemia were attributable to iron and vitamin A deficiencies, respectively, whereas household possession of soap was associated with approximately 11% fewer cases of anaemia. ID in children was associated with vitamin A deficiency and stunting, whereas inflammation was associated with iron sufficiency. Among women, 40.3% were anaemic, and 49.7% were iron deficient. In women, ID and number of births were significantly associated with anaemia in multivariate models, and approximately 42% of anaemia in women was attributable to ID. Increased parity was associated with ID, and incubation and early convalescent inflammation was associated with ID, whereas late convalescent inflammation was associated with iron sufficiency. ID is the main risk factor of anaemia in both women and children and contributed to a substantial portion of the anaemia cases. To tackle both anaemia and ID in Somalia, food assistance and micronutrient‐specific programmes (e.g. micronutrient powders and iron supplements) should be enhanced.

**Key messages**

- The prevalence of anaemia is classified as a severe public health problem in Somali children and women, and the iron deficiency prevalence is considered high and moderate for children and women, respectively.
- Iron deficiency is the main risk factor of anaemia in both children and women; however, the risk factors of iron deficiency are not the same for each group.
- Programme planners can likely reduce the prevalence of anaemia and iron deficiency by increasing the coverage of nutrition interventions that increase the intake of iron and vitamin A, such as the distribution of MNPs, micronutrient supplements and vitamin A supplements.

## 1 INTRODUCTION

Anaemia is characterized by a shortage of functional haemoglobin (Kassebaum et al., 2016). It is estimated that anaemia affects approximately one‐quarter of the world's population and is more prevalent in young children and women of reproductive age (Stevens et al., 2013). Anaemia has serious health consequences and significantly contributes to the global burden of disease (Kassebaum et al., 2014; WHO, 2004).

Biological parameters (e.g. age, gender and pregnancy) as well as external household‐level factors (e.g. household wealth, water quality and sanitation facility) have been associated with anaemia in children and women (WHO, 2017). The causes of anaemia are multifactorial, sometimes even in the same person, and anaemia is caused by nutritional factors, such as iron, vitamin A, folate and vitamin B_12_ deficiencies; acute and chronic inflammation; parasitic infections; and blood disorders (WHO, 2011a). Globally, iron deficiency (ID) has been identified as the principal risk factor for anaemia (Kassebaum et al., 2016); however, there are some locales where other risk factors (e.g. malaria) make a larger contribution to the overall anaemia burden (Kassebaum et al., 2014). In sub‐Saharan African countries that have a high prevalence of anaemia and a very high burden of infection, the proportion of anaemia associated with ID has been shown to be relatively small (Petry et al., 2016). In addition to being a risk factor of anaemia, ID has direct sequelae, such as cognitive impairment (Jáuregui‐Lobera, 2014) and reduced work productivity (Haas & Brownlie, 2001).

Awareness of anaemia and ID and interventions to address both conditions have increased over the past decades (Stevens et al., 2013). However, before national interventions and programmes can be effectively implemented, the prevalence and severity of anaemia together with its underlying risk factors should be assessed.

Data on anaemia and ID in Somali children and women of reproductive age was assessed more than a decade ago as part of a national survey in 2009 (FSNAU, FAO, & UCL, 2009). The results of this survey indicated that anaemia was a severe public health problem (WHO, 2011a) and iron depletion was prevalent (WHO, 2011b) according to WHO criteria. Although this survey assessed many of the main anaemia risk factors, such as ID, vitamin A deficiency, recent malaria and acute infection, the contribution of each of these factors to the overall anaemia burden was not analysed.

Moreover, over the past decade, Somalia has suffered from multiple recurring natural and manmade emergencies, including political instability, drought, famine and floods; these events led to internal displacement and the deterioration of household food security throughout Somalia (UNDP, 2018). In addition, in the past decade, some public health programmes that could affect anaemia and ID were established. Although there are scant data on the coverage of iron/folate supplementation, home fortification and deworming, a 2014/2015 survey found that more than 50% of children 6–59 months of age receive vitamin A supplements in the past 6 months (FSNAU, 2015).

The 2019 Somalia Micronutrient Survey (SMS 2019) collected information about anaemia prevalence, ID and other micronutrient deficiencies and data about risk factors for anaemia and ID in order to measure the relative importance of several potential risk factors for anaemia and ID.

## 2 METHODS

### 2.1 Survey design and participants

The SMS 2019 was a nationwide cross‐sectional stratified survey based on a probability sample. It was designed to produce estimates that have acceptable precision for priority indicators of nutritional status in children 0–59 months of age (6–59 months of age for blood biomarkers) and women of reproductive age. Data were collected between December 2018 and September 2019. The survey sampled households separately in the following geographic strata: (1) Somaliland, (2) Puntland, (3) Hirshabelle and Galmudug states, (4) Jubaland and South West states and (5) Banaadir region. In addition, a sixth stratum consisted of internally displaced person (IDP) settlements in all of Somalia. Two‐stage sampling was conducted. In the first stage, enumeration areas listed as part of the 2014 Population Estimation Survey of Somalia (United Nations Population Fund, 2014) served as the primary sampling units (PSUs) and were selected with probability proportional to size methodology for the five *geographic* strata. For the IDP stratum, IDP settlements identified by the United Nations High Commissioner for Refugees (UNHCR, 2018) in 2018 served as PSUs and were also selected probability proportional to size. For all strata, PSUs that were not accessible due to security reasons were excluded from the sampling frame prior to random selection. Each stratum contained 25 PSUs, with 150 PSUs selected in total. In the second sampling stage, 16 households were randomly selected from each PSU using simple random sampling after survey teams made an updated list of all households residing within the PSU boundaries just prior interviewing. In total, 2400 households were randomly selected, and data were successfully collected from 2172 households (90.5% household response rate). In each consenting household, survey teams attempted to recruit all children 0–59 months. Non‐pregnant women of reproductive age (15–49 years) were recruited only in every second selected household.

### 2.2 Data collection and laboratory analysis

Prior to data collection, multiple trainings were conducted for team leaders, interviewers, anthropometrists and phlebotomists to ensure that all fieldworkers were entirely competent in the various data collection procedures. In addition, all procedures were field‐tested in practice PSUs prior to the start of fieldwork. Further details of the survey training are described elsewhere (Federal Ministry of Health et al., 2020).

During the fieldwork, interviewers began data collection at consenting households by first administering the household questionnaire to the head of the household or another knowledgeable adult household member. Following the completion of the household questionnaire, interviewers administered the child questionnaire to the caretakers of all children 0–59 months of age and the woman questionnaire to non‐pregnant women in every second household. The child questionnaire was used to collect information on child age, sex, recent illness and consumption of supplements and fortified food products. For woman, questionnaires were used to collect information about age, educational achievement, occupation, marital and pregnancy status, supplement consumption and dietary diversity.

A capillary blood sample was collected via finger prick from non‐pregnant women and children 12–59 months of age; blood was collected via heel prick from children 6 to 11 months of age. Prior to lancet puncture, the finger or heel was disinfected with an alcohol pad and wiped dry with a sterile gauze pad. Following the lancet puncture (BD®, high‐flow blade 1.5 × 2.0 mm lancet), the first blood drop was discarded, the second blood drop was used to measure malaria parasitemia with a rapid test kit (SD Bioline, Malaria Ag P.f/Pan), and the third drop was used to measure haemoglobin concentration using HemoCue™ (Hb301+, HemoCue™, Ängelholm, Sweden). Quality control blood of low and medium haemoglobin concentration (Eurotrol, Ede, Netherlands) was used on a daily basis to assure accurate haemoglobin measurements with the HemoCue™ devices. Following the on‐site measurements, 300–400 μl of blood was collected from children and non‐pregnant women into a silica‐coated blood collection tube (Sarstedt, Microvette® 300 Z).

Whole blood samples were stored in cold boxes at 4–8°C until further processing later the same day. Whole blood samples were centrifuged at 3000 rpm for 7 min to separate the serum using mobile centrifuges (Steinberg Systems, Berlin, Germany). Serum was then aliquoted into appropriately labelled cryovials and stored in portable freezers at −20°C (Prime Tech, Neuwied, Germany) or stationary freezers (where possible) for the duration of the field data collection. After completion of fieldwork, all blood samples were transferred to a central freezer at Hargeisa, Somaliland, before shipment with frozen saltwater cold packs to international laboratories for analyses. All samples arrived frozen at their respective destinations.

Serum specimens from children and women were analysed for retinol‐binding protein (RBP), ferritin, C‐reactive protein (CRP) and α1‐acid glycoprotein (AGP) at the VitMin Lab (Wilstätt, Germany) using an ELISA method (Erhardt et al., 2004). Serum zinc was analysed in a subsample of children at the University of California, Davis, USA, using inductively coupled plasma mass spectrometry. Serum folate and vitamin B_12_ were analysed in a subsample of women by the Swiss Vitamin Institute (Epalinges, Switzerland) using microbiological assays with *Lactobacillus casei* (*ATCC 7469*) and *Lactobacillus leichmannii* (*ATCC 7830*) as test organisms, respectively, following the turbidimetric reference method performed in microtitre plates (O'Broin et al., 1997). Both VitMin Lab and the Swiss Vitamin Institute participate in the Vitamin A Laboratory – External Quality Assurance (VITAL‐EQA) programme (US CDC, 2021) and demonstrated minimal imprecision and bias in the VITAL‐EQA rounds prior to analysing samples from the SMS 2019.

In children 6–59 months of age, polymerase chain reaction was used to test for sickle cell disease (HbSS), sickle cell trait (HbAS), homozygous and heterozygous α‐thalassaemia and the homozygous/hemizygous and heterozygous G6PD c.202 form of glucose‐6‐phosphate dehydrogenase (G6PD) deficiency. Genetic analyses were conducted at the KEMRI‐Wellcome Trust Research Programme in Kilifi, Kenya (Atkinson et al., 2006; Chong et al., 2000; Waterfall & Cobb, 2001). These genetic blood conditions affect the shape (sickle cell disease and trait and α‐thalassaemia) (Farashi & Harteveld, 2018; WHO, 2006) and the stability (G6PD deficiency) (Luzzatto et al., 2016) of red blood cells and can potentially affect an individual's haemoglobin concentration.

### 2.3 Parameters and clinical thresholds

After adjusting haemoglobin for altitude according to WHO guidelines, anaemia in children was defined as a haemoglobin concentration less than 110 g/L, and haemoglobin concentrations of <70, 70–99 and 100–109 g/L were used to define severe, moderate and mild anaemia, respectively. For non‐pregnant women, anaemia was defined as a haemoglobin concentration of less than 120 g/L. Haemoglobin concentrations of <80, 80–109 and 110–119 g/L denoted severe, moderate and mild anaemia, respectively (WHO, 2011a). No adjustment to women's haemoglobin concentration was made based on their tobacco smoking habits; smoking is very rare in Somalia, thus, no information was collected on smoking practices.

As serum ferritin and RBP are acute‐phase proteins, their concentrations are elevated and decreased, respectively, in the presence of infection or other causes of inflammation. To account for these infection‐related changes in blood proteins, serum ferritin and RBP concentrations were adjusted for inflammation using the correction algorithm developed by the BRINDA project (Larson et al., 2017; Namaste et al., 2017). In brief, the BRINDA correction algorithm applies a continuous adjustment based on the linear associations between concentrations of inflammation biomarkers (i.e. CRP and AGP) and ferritin and RBP. Inflammation‐adjusted serum ferritin concentrations of <12 and <15 μg/L defined ID in children and non‐pregnant women, respectively (WHO, 2011b). As recommended by the BRINDA project, RBP was not adjusted in women (Larson et al., 2017). Thus, vitamin A deficiency was defined as RBP concentrations <0.7 μM/L (WHO, 2011c) using inflammation‐adjusted RBP concentrations in children and using unadjusted RBP concentrations in women.

Concentrations of >5 mg/L and >1 g/L were used as cut‐offs for elevated CRP and AGP, respectively. Subclinical inflammation was categorized into incubation (elevated CRP only), early convalescence (elevated CRP and AGP) and late convalescence (elevated AGP only) according to Thurnham et al. (2008). To classify a child as zinc deficient, two thresholds were used depending on the time of day when the blood sample was collected: non‐fasting serum zinc <65 (morning) and <57 μg/dl (afternoon) (King et al., 2016). In women, concentration of serum folate < 10 nmol/L denoted folate deficiency, and concentrations of serum B_12_ < 150 pmol/L denoted vitamin B_12_ deficiency (WHO & FAO, 2006).

Child stunting, wasting, underweight, overweight and obesity were assessed using the WHO Growth Standards (WHO Multicentre Growth Reference Study Group, 2006). Height‐for‐age, weight‐for‐height and weight‐for‐age z‐scores < −2.0 defined stunting, wasting or underweight, respectively. Overweight in children was defined as a weight‐for‐height z‐score > + 2.0 but ≤+3.0; obesity was defined as a weight‐for‐height z‐score > + 3.0.

Chronic energy deficiency and overnutrition in non‐pregnant women were defined based on body mass index (BMI; kg/m^2^). Cut‐offs for BMI were as follows: <16.0 severe chronic energy deficiency; 16.0–16.9 moderate chronic energy deficiency; 17.0–18.4 at‐risk for energy deficiency; 18.5–24.9 normal; 25.0–29.9 overweight; ≥30.0 obese (Shetty & James, 1994).

### 2.4 Data management and statistical analysis

Data were electronically collected using Open Data Kit (ODK) software on tablet computers. To ensure data validity and integrity, multiple steps were taken. Firstly, as the SMS 2019 administered three separate questionnaires (i.e. household, child and woman), the ODK questionnaires were programmed to facilitate the merging of household‐level data with individual‐level data. To illustrate, one ODK questionnaire form was composed of many individual questionnaires, such that the data for all children or women residing in a household were collected as part of the same form. Secondly, during the data collection, summary statistics of key variables were calculated for each team to identify any possible data collection errors. When potential issues were identified, teams were contacted to clarify the issue. Thirdly, any possible discrepancies found during the data analysis were cross‐checked against the paper biological forms that were collected for each child and woman. Data analysis was done using Stata/IC Version 14.2 (StataCorp., Texas, USA). To account for unequal probability of selection in the six strata, statistical weights were applied in all analyses, which combined two or more strata.

The statistical precision of prevalence estimations was assessed using 95% confidence intervals (95% CI), which were calculated accounting for the complex sampling. Only variables that were assessed in all subjects and not only in a subsample were considered for inclusion into multivariable models.

Weighted means and CI were calculated for normally distributed continuous variables. For non‐normally distributed continuous variables, weighted medians and CI were calculated using the methodology of Francisco and Fuller (2007) via Stata's *epctile* command (Kolenikov, n.d.). For categorical variables, the precision of weighted prevalence rates in each subgroup was indicated with 95% CI. Adjusted chi‐square was used to measure the statistical precision of apparent differences between subgroups.

Bivariate analysis was done for potential risk factors of anaemia and ID. Variables for which the *P*‐value < 0.1 for the association between that variable and either anaemia or ID were included in multivariable models.

Prior to running the multivariable regression models, covariate colinearity was examined using the variance inflation factor (VIF), and colinear variables were identified and removed accordingly. The remaining variables were included into a Poisson regression model with robust error variance; non‐significant variables were manually removed using backward elimination until all variables were statistically significant (i.e. *P* < 0.05). Using the adjusted risk ratios produced by the Poisson regression and the prevalence of exposure to the risk factor among anaemic children or women, we calculated the population attributable fraction (PAF) for each risk factor retained in the final regression models (Rockhill et al., 1998).

### 2.5 Ethical considerations

Ethical permissions for the survey were obtained from the ethical review committees of the Federal Ministry of Health and the Ministries of Health in Somaliland and Puntland. In addition, written consent was obtained for the administration of the household questionnaire from the household head or another knowledgeable household member. This written consent included permission to request verbal consent from all participating non‐pregnant women 15–49 years and from the caretakers of children 6–59 months of age. If consenting individuals were illiterate, the consent form was read out loud to them, and a fingerprint was taken as evidence of consent instead of a signature.

If diagnosed with malaria, severe acute malnutrition and/or severe anaemia, survey participants were referred to local health facilities for treatment. No blood was taken from children younger than 6 months of age to avoid injury.

## 3 RESULTS

Basic demographic information on the survey respondents and the results of the anthropometric measurements (e.g. stunting, wasting, underweight, overweight and obesity prevalence) are presented elsewhere (Federal Ministry of Health et al., 2020). As shown in Table 1, almost half of the children were anaemic; most had mild or moderate anaemia. ID and iron deficiency anaemia (IDA) were found in approximately one‐half and one‐quarter of children, respectively. Both the anaemia (WHO, 2011a) and ID prevalence (WHO, 2020a) in children are considered a severe public health problem according to WHO classifications. More than one‐third of children were deficient in vitamin A; this prevalence also indicates a severe public health problem according to WHO classifications (WHO, 2011c). Approximately one‐quarter of children were found to have subclinical inflammation, most being in the late convalescence phase. The prevalence in children of the investigated blood disorders was relatively low: Sickle cell trait and disease were almost non‐existent, and very few children had heterozygous/hemizygous or homozygous G6PD deficiency. α‐Thalassaemia was found in 7% of children, nearly all cases being heterozygotes. Malaria was rare; only 0.5% (95% CI: 0.2, 1.1) of children tested positive.

**Table 1:** Anaemia, inflammation and micronutrient indicators of preschool children 6–59 months of age, Somalia 2019

Characteristic	N	% ^a^/mean/median	95% CI^a^
Children 6–59 months			
Haemoglobin indicators			
Haemoglobin (g/L), mean	1,667	110.3	(109.0, 111.6)
Any anaemia^b^, %	1,667	43.4	(40.0, 46.9)
Mild anaemia^b^, %	361	21.4	(19.3, 23.6)
Moderate anaemia^b^, %	342	20.5	(18.1, 23.2)
Severe anemia^b^, %	29	1.5	(0.9, 2.5)
Iron status^c^ ^,^ ^d^			
Ferritin (μg/L), median, unadjusted	1,480	15.0	(12.9, 17.1)
Ferritin (μg/L), median, adjusted	1,480	13.4	(11.5, 15.4)
Iron deficiency, %	1,480	47.2	(43.4, 51.1)
Iron deficiency anaemia, %	1,463	28.6	(25.5, 31.9)
Vitamin A status^c^ ^,^ ^e^			
RBP (μmol/L), mean, unadjusted	1,480	0.80	(0.77, 0.83)
RBP (μmol/L), mean, adjusted	1,480	0.83	(0.79,0.86)
Vitamin A deficiency	1,480	34.4	(31.4, 37.5)
Zinc status			
Serum zinc (μg/dL), mean	142	102.7	(95.2, 110.3)
Zinc deficiency^g^, %	142	5.0	(2.2, 11.2)
Inflammation status			
No inflammation, %	1,104	74.7	(71.6, 77.5)
Any inflammation^f^, %	1,480	25.3	(22.5, 28.4)
Elevated CRP only, %	47	3.0	(1.9, 4.5)
Elevated CRP and AGP, %	105	7.2	(5.8, 8.9)
Elevated AGP only, %	224	15.2	(12.8, 17.9)
Sickle cell status			
Normal (HbAA), %	1,481	99.3	(98.5, 99.7)
Sickle cell trait (HbAS), %	10	0.7	(0.3, 1.4)
Sickle cell disease (HbSS), %	1	0	(0, 0.3)
α‐Thalassaemia status			
No α‐thalassaemia deletions, %	1,331	92.5	(90.1, 94.3)
Heterozygous α‐thalassaemia, %	101	7.0	(5.3, 9.3)
Homozygous α‐thalassaemia, %	9	0.5	(0.2, 1.2)
G6PD status			
No G6PD deficiency, %	1,447	97.2	(95.8, 98.1)
Heterozygous G6PD deficiency, %	24	1.4	(0.9, 2.3)
Homo‐/hemizygous G6PD deficiency, %	20	1.4	(0.9, 2.3)

*Note:* The n's are unweighted numbers in each subgroup; subgroups that do not sum to the total have missing data.

Abbreviation: CI, confidence interval.

^a^ Calculated taking into account the complex sampling design.

^b^ Anaemia in children defined in as Hb < 110 g/L; severe, moderate and mild anaemia defined as haemoglobin <70, 70–99 and 100–109, respectively. Haemoglobin concentrations were adjusted for altitude.

^c^ Ferritin and RBP concentrations and associated deficiency prevalences corrected for inflammation according to BRINDA (Larson et al., 2017; Namaste et al., 2017).

^d^ Iron deficiency defined as serum ferritin < 12 μg/L in children and serum ferritin < 15 μg/L in women; iron deficiency anaemia defined as low serum ferritin and low haemoglobin.

^e^ Vitamin A deficiency defined as RBP < 0.7 μmol/L.

^f^ Any inflammation defined as elevated CRP, elevated AGP or elevated CRP and AGP.

^g^ Percentages weighted for unequal probability of selection.

^h^ Zinc deficiency defined as non‐fasting serum zinc < 65 or <57 μg/dL depending on if blood was collected in the morning or afternoon, respectively (King et al., 2016).

As shown in Table 2, anaemia was found in approximately 40% of non‐pregnant women, with most anaemia being either mild or moderate. According to WHO classifications (WHO, 2011a), anaemia in women is a severe public health problem. ID was found in nearly one‐half of women, and IDA found in nearly one‐third. Approximately 10% of women were vitamin A deficient, and about one‐third of the women had subclinical inflammation. Nearly half of women with inflammation had elevated CRP only (incubation phase). Folate and vitamin B_12_ deficiencies were found in more than one‐third of women, but these indicators were only measured in a subsample of about 15% of women. Malaria was also rare in women; only 0.3% (95% CI: 0.1, 1.4) of non‐pregnant women tested positive.

**Table 2:** Anaemia, inflammation and micronutrient indicators of women of reproductive age, Somalia 2019

Characteristic	*N*	% ^a^/mean/median	95% CI^a^
Non‐pregnant women			
Haemoglobin concentration			
Haemoglobin (g/L), mean	777	121.4	(119.8, 122.9)
Any anaemia^b^, %	333	40.3	(36.2, 44.4)
Mild anaemia^b^, %	155	19.7	(16.8, 23)
Moderate anaemia^b^, %	159	18.0	(15.4, 20.9)
Severe anemia^b^,%	19	2.6	(1.6, 4.1)
Iron status^c^ ^,^ ^d^			
Ferritin (μg/L), median, unadjusted	687	17.9	(15.5, 20.3)
Ferritin (μg/L), median, adjusted	687	15.1	(13.0, 17.3)
Iron deficiency, %	687	49.7	(45.4, 54.0)
Iron deficiency anaemia, %	680	27.8	(24.2, 31.7)
Vitamin A status^c^ ^,^ ^e^			
RBP (μmol/L), mean, unadjusted	687	1.08	(1.05, 1.13)
Vitamin A deficiency, %	687	10.7	(8.4, 13.5)
Folate status^f^			
Serum folate (nmol/L), mean	141	13.5	(12.4, 14.7)
Folate deficiency, %	141	35.1	(26.7, 44.7)
Vitamin B_12_ status^g^			
Serum B_12_ (pmol/L), median	128	180.3	(139.3, 221.2)
Vitamin B_12_ deficiency, %	128	36.9	(25.4, 50.1)
Inflammation status			
No inflammation, %	455	66.1	(62.4, 69.7)
Any inflammation^h^, %	687	33.9	(30.3, 37.6)
Elevated CRP only, %	108	15.1	(12.7, 18)
Elevated CRP and AGP, %	88	12.9	(10.8, 15.4)
Elevated AGP only, %	36	5.8	(4.1, 8.2)

*Note:* The n's are unweighted numbers in each subgroup; subgroups that do not sum to the total have missing data.

Abbreviation: CI, confidence interval.

^a^ Calculated taking into account the complex sampling design.

^b^ Anaemia in children defined in as Hb < 110 g/L; severe, moderate and mild anaemia defined as haemoglobin <70, 70–99 and 100–109, respectively; in women anaemia defined as Hb < 120 g/L; severe, moderate and mild anaemia as haemoglobin <80, 80–109 and 109–120, respectively.

^c^ Ferritin concentrations and ID prevalence was corrected for inflammation according to BRINDA (Namaste et al., 2017).

^d^ Iron deficiency defined as serum ferritin < 12 μg/L in children and serum ferritin < 15 μg/L in women; iron deficiency anaemia defined as low serum ferritin and low haemoglobin.

^e^ Vitamin A deficiency defined as RBP < 0.7 μmol/L.

^f^ Folate deficiency defined as serum folate < 10 nmol/L (WHO & FAO, 2006).

^g^ Vitamin B_12_ deficiency defined as serum B_12_ < 150 pmol/L (WHO & FAO, 2006).

^h^ Any inflammation defined as elevated CRP, elevated AGP or elevated CRP and AGP.

^i^ Percentages weighted for unequal probability of selection.

Tables S1 and S2 show the bivariate analysis of various potential risk factors of anaemia in children and women, respectively. In children, several individual‐level variables were associated with anaemia status with a *P*‐value < 0.1, including ID, vitamin A deficiency, stunting, inflammation, α‐thalassaemia, G6PD deficiency, child weighed at birth, child received vitamin A supplements in the past 6 months and child registered in a feeding programme. Household‐level variables associated with anaemia in children included household wealth quintile, household food insecurity category and any soap in the house. In women, fewer variables were associated with anaemia status; individual‐level variables included ID, vitamin A deficiency and marital status, and household‐level variables included water at household's handwashing site and household wealth quintile.

Tables S3 and S4 show the bivariate analysis of various potential risk factors of ID status in children and women, respectively. In children, the individual‐level variables associated (*P*‐value < 0.1) with ID included sex, vitamin A deficiency, inflammation, stunting, wasting and consumed fortified cereal in the past 24 h. State of residence was the only non‐individual‐level variable associated with ID in children. In women, individual‐level variables associated with ID status included pregnancy in the past 2 years, inflammation and consumed organ meat in past 24 h. Household wealth quintile was the only household‐level variable associated with ID in women.

Age can greatly impact a child's diet and interaction with his/her environment; therefore, the associations between age in months—a continuous variable—and anaemia and ID were examined separately using linear regression analysis. Age in months was significantly associated with anaemia (coefficient = 0.71; *P* < 0.001) and ID (coefficient = 0.70; *P* < 0.001). Age category in months was also significantly associated with anaemia status (see Table S1) and ID (see Table S3). Age in months as a continuous variable was included in both anaemia and ID regression models for children to increase the model's accuracy.

Colinearity was examined prior to developing parsimonious regression models. In the initial anaemia regression models, no colinearity was observed, and all independent variables had a VIF < 1.2 and VIF < 1.5 in the child and women models, respectively. During the construction of the anaemia models for children and women, backward elimination was used to exclude non‐significant factors one at a time. Only a small number of risk factors were retained in the final regression models.

As shown in Table 3, the most important risk factor for anaemia in children was ID, which accounted for more than one‐third of cases. Other factors, such as vitamin A deficiency, α‐thalassaemia and inflammation, accounted for a much smaller proportion of cases of anaemia. Having soap in the child's household was protective against anaemia. Household wealth quintile did not substantially contribute to anaemia.

**Table 3:** Adjusted relative risk of anaemia in children 6–59 months and non‐pregnant women 15–49 years of age, Somalia 2019

Characteristic	Category	Adjusted relative risk	95% CI	Sig.^a^	Population attributable fraction^b^
Children 6–59 months^c^		*N* = 1456			
Iron status	Deficient	2.15	(1.87, 2.47)	^***^	36.2%
	Not deficient	Referent			
Vitamin A status	Deficient	1.17	(1.04, 1.31)	^*^	5.6%
	Not deficient	Referent			
α‐Thalassaemia	Hetero‐/homozygous	1.32	(1.07, 1.57)	^**^	2.2%
	None	Referent			
Inflammation	Yes	1.15	(1.02, 1.30)	^*^	4.2%
	No	Referent			
Household has soap	Yes	0.84	(0.74, 0.95)	^**^	−10.8%
	No	Referent			
Wealth quintile	Lowest	Referent			
	Second	0.88	(0.71, 1.10)		−2.6%
	Middle	0.87	(0.70, 1.08)		−3.3%
	Fourth	0.88	(0.71, 1.09)		−3.2%
	Highest	0.71	(0.56, 0.91)	^**^	−7.6%
Non‐pregnant women 15–49 years		*N* = 583			
Iron status	Deficient	2.44	(1.97, 3.00)	^***^	42.3%
	Not deficient	Referent			
Number of births	Never pregnant	Referent			
	0–1 birth	1.33	(0.98, 1.81)		6.4%
	2–3 births	1.43	(1.07, 1.90)	^*^	6.9%
	4–5 births	1.22	(0.89, 1.68)		2.7%
	6–7 births	1.49	(1.09, 2.05)	^*^	4.9%
	8+ births	1.57	(1.12, 2.21)	^*^	2.7%

^a^ Statistically significant associations of each subgroup with anaemia.

^b^ Calculated using relative risk from Poisson regression and the proportion of anaemia among exposed (e.g. iron deficient) individuals.

^c^ Child's age in months as a continuous covariate was included in the child regression model.

^*^ *P* < 0.05.

^**^ *P* < 0.01.

^***^ *P* < 0.001.

In women, the risk factor making the greatest contribution to anaemia was ID. The number of pregnancies was also associated with anaemia, with a significantly increased risk of anaemia found in women that have previously had 2–3, 6–7 or ≥8 births. Although the PAF of anaemia attributable to births was minimal for each category, the three aforementioned categories contributed to nearly 15% of all anaemia cases in women.

The results of multivariable analyses of risk factors for ID are shown in Table 4. In the initial ID regression models, no colinearity was observed, and all independent variables had a VIF < 1.1 and VIF < 1.5 in the child and women models, respectively. Children with vitamin A deficiency or stunting have about 30% and 40% increased risk of being iron deficient, respectively. Nonetheless, only about 16% of ID was attributable to vitamin A deficiency and stunting. All types of inflammation were independently associated with lower risks of ID in children, but the attributable reduction of ID to inflammation was only marginal.

**Table 4:** Adjusted relative risk of iron deficiency in children 6–59 months and non‐pregnant women 15–49 years of age, Somalia 2019

Characteristic	Category	Adjusted relative risk	95% CI	Sig.^a^	Population attributable fraction^b^
Children 6–59 months^c^		*N* = 1456			
Vitamin A status	Deficient	1.30	(1.16, 1.46)	^***^	9.4%
	Not deficient	Referent			
Inflammation	Elevated CRP, only	0.56	(0.36, 0.88)	^*^	−1.6%
	Elevated CRP and AGP	0.65	(0.50; 0.86)	^**^	−2.9%
	Elevated AGP, only	0.85	(0.73; 0.99)	^*^	−2.9%
	None	Referent			
Stunting	Yes	1.44	(1.27, 1.62)	^***^	7.1%
	No	Referent			
Non‐pregnant women 15–49 years		*N* = 680			
Any soap in HH	Yes	1.53	(1.26, 1.87)	^***^	23.8%
	No	Referent			
Inflammation	Elevated CRP, only	0.86	(0.69, 1.09)		−2.2%
	Elevated CRP and AGP	0.67	(0.49, 0.91)	^*^	−4.9%
	Elevated AGP, only	1.33	(1.04, 1.70)	^*^	2.0%
	None	Referent			
Number of births	Never pregnant	Referent			
	1 birth	1.30	(0.99, 1.71)		5.3%
	2–3 births	1.47	(1.14, 1.90)	^***^	6.7%
	4–5 births	1.39	(1.07, 1.80)	^*^	5.0%
	6–7 births	1.52	(1.15, 2.03)	^**^	5.1%
	8+ births	1.33	(0.95, 1.86)		1.8%

^a^ Statistically significant associations of each subgroup with iron deficiency.

^b^ Calculated using relative risk from Poisson regression and the proportion of anaemia among exposed (e.g. iron deficient) individuals.

^c^ Child's age in months as a continuous covariate was included in the child regression model.

^*^ *P* < 0.05.

^**^ *P* < 0.01.

^***^ *P* < 0.001.

In women, a higher risk of ID was found in those residing in households that possessed soap, and more than 20% of ID was attributable to possession of soap. Number of births was also associated with ID, with significant associations with ID found for women that had 2–3, 4–5 and 6–7 births. Nearly 17% of ID cases were attributable to these three subgroups.

## 4 DISCUSSION

### 4.1 Risk factors of anaemia and ID

#### 4.1.1 Iron and vitamin A deficiencies

ID was identified as the main anaemia risk factor in children and women in Somalia. These findings are not surprising considering the fact that the prevalence rates of other major anaemia risk factors such as malaria and inflammation were relatively low. Moreover, the PAF of anaemia attributable to ID in Somalia is similar to that found in the Gambia (Petry et al., 2019), which also had a low malaria prevalence and utilized the same methodology of calculating PAF.

In children, the multivariable models showed that vitamin A deficiency is also associated with anaemia. We also identified vitamin A deficiency as a risk factor for ID in children. Although both ID and vitamin A deficiency may be coincident results of other risk factors, such as a poor diet with low micronutrient intake, vitamin A deficiency may directly contribute to anaemia by reducing erythropoiesis and contribute to ID by trapping iron in the liver and thus reducing serum iron concentration (Bloem et al., 1989; Semba & Bloem, 2002). Regarding the association between vitamin A deficiency and ID in children, the direction of this association is uncertain as iron‐deficient children could also have been deficient in vitamin A due to generally poor‐quality diets. However, this association may indicate that vitamin A deficiency, which can limit the mobilization of iron stores, results in increased levels of iron sequestration (Zimmermann et al., 2006).

#### 4.1.2 Inflammation and malaria

Inflammation was associated with both anaemia and ID in children. The prevalence of inflammation observed in children was markedly lower than that found in other surveys conducted in sub‐Saharan Africa (Engle‐Stone et al., 2017; University of Ghana et al., 2017; Wirth et al., 2016). This relatively low prevalence explains in part why inflammation did not contribute substantially to the PAF of anaemia or ID in children. Inflammation has been previously identified as a risk factor of anaemia, as chronic inflammation suppresses erythropoiesis, reduces the ‘lifespan’ of erythrocytes and elevates levels of hepcidin, which inhibits the absorption of iron (Nemeth & Ganz, 2014). Although inflammation is not a risk factor of ID, serum ferritin is an acute‐phase protein that increases during periods of inflammation (Namaste et al., 2017). Although the serum ferritin values were adjusted for the presence of inflammation (Namaste et al., 2017), such adjustment may be somewhat incomplete in children. This would result in an apparent association of inflammation with iron sufficiency due to higher ferritin concentrations. In addition, incomplete adjustment for inflammation would result in an underestimation of the prevalence of ID.

In women, the associations between inflammation and ID were mixed, with women in the early convalescence phase having a lower risk of ID and those in the late convalescent phase having a higher risk of ID. But a higher risk of ID in the late convalescent phase (elevated AGP only) can be explained by the hepcidin‐induced blockage of iron absorption in the state of inflammation (Girelli et al., 2016; Wang & Babitt, 2016). The lower risk of ID in the early convalescence phase (elevated AGP and CRP), however, is not clear. These mixed associations demonstrate the linkage between iron status and inflammation and also complex relationship between the two conditions.

In addition to inflammation, malaria can trigger anaemia and—along with inflammation—is one of main types of non‐nutritionally induced anaemia (WHO, 2017). The majority of fieldwork for this survey was conducted during dry seasons, and as such, the malaria prevalence and its association between anaemia and ID may be underestimated. In addition, some malaria endemic areas (Federal Government of Somalia, Government of Puntland & Government of Somaliland, 2016) were not included in the sample frame of the SMS 2019 due to high levels of insecurity, and this may contribute to the low malaria prevalence observed and may have also resulted in an underestimation of the anaemia prevalence in children and women. Regardless, due to the low prevalence measured using rapid diagnostic tests, public health officials in Somalia can consider more sensitive methods for detecting asymptomatic malaria infection, such as molecular methods that measure the presence of malaria antigens (Hofmann et al., 2015). Identification and treatment of malaria infection during the dry seasons (when malaria infection is lowest) is key to eradicate malaria (Moiroux et al., 2012), as underlying malaria infections can contribute in higher malaria transmission rates during rainy seasons where mosquitoes and parasite development rates are highest (Najera et al., 1998).

#### 4.1.3 Stunting

The association between ID and stunting was observed in children and likely captures the fact that both conditions are caused by poor diets and/or malnutrition. Fançony et al. (2020) found an association between stunting and IDA in northern Angola and speculated that ‘long periods of nutritional restriction’ is potentially the main driver of both IDA and stunting.

#### 4.1.4 Parity

Number of births was significantly associated with higher anaemia and ID prevalence in both multivariable models for women. Similar associations between anaemia and increased parity have been found in other countries (Imai, 2020; Mei et al., 2011; Shamah‐Levy et al., 2009), and our findings may suggest that increased parity in Somalia, and potentially short intervals between births, contributes to anaemia and ID. The association between the number of births and anaemia or ID likely reflects the reduced ferritin levels that occurred during pregnancy from increased iron requirements and the ID post‐partum attributable to blood loss (Breymann et al., 2017). Both conditions subsequently result in lower haemoglobin levels and increased risk of anaemia (Breymann, 2015). Blood loss due to post‐partum haemorrhage can also contribute lower ferritin and haemoglobin concentrations (Anger et al., 2019).

Although there was a significant association between ID and women that had between two and seven births, there was no significant association between ID and women that had only one birth. This finding may suggest that women that had only one delivery were able to replenish their iron stores more readily than women with ≥2 deliveries. In a study comparing the iron status of multiparous and nulliparous women during pregnancy, Imai (2020) found lower ferritin levels in the first trimester of multiparous women, suggesting that women beginning their second or greater pregnancy had not yet fully regained their iron stores from the losses that occurred during previous pregnancies and deliveries. It is unclear why no significant association was observed between ID and women with eight or more pregnancies.

#### 4.1.5 Household‐level factors

Only two household‐level factors were retained in multivariable models: possession of soap and household wealth quintile. Regarding soap possession, poor sanitary conditions and the exposure to faecal–oral pathogens have been shown to be related to inflammation in developing countries (Cumming & Cairncross, 2016; McKay et al., 2010) and might be a plausible underlying anaemia risk factor. Our data suggest that the possession of soap may have a protective effect against anaemia in children. Although the 2019 SMS did not collect data on handwashing or bathing practices, this association could stem from improved hygiene from handwashing and bathing of children. Handwashing has been shown to reduce anaemia in school‐age children in Ethiopia (Mahmud et al., 2015); however, other studies have found no impact of improved hygiene practices (including soap) on anaemia in young children (Humphrey et al., 2019). In contrast, household soap ownership was associated with an increased risk of ID in women. This finding is counter‐intuitive and is difficult to explain. This finding may be a result of confounding by an unmeasured variable, or it could be an entirely spurious finding resulting from sampling error.

Our study found that household wealth was associated with anaemia in children. However, only children residing in the households within the highest wealth quintile had a significantly lower risk of anaemia compared with the children in the lowest wealth quintile. This finding may suggest that children from households in the highest wealth quintile consume a superior diet than children in the bottom four quintiles, or alternatively, that children living in households in the highest wealth quintile have a limited exposure to environmental risk factors of anaemia (e.g. helminths).

### 4.2 Possible programmatic response

The high prevalence of iron and vitamin A deficiencies in children and women—and their association with anaemia (vitamin A deficiency in children only)—highlights the need to increase the intake of bioavailable iron and vitamin A in Somalia. Although relatively few children and women consume a diet with minimum diversity, promotional campaigns to increase the quantity and variety of foods consumed may not currently be a viable programme option in Somalia. Recent research from the World Food Programme (WFP) (World Food Programme, Scaling Up Nutrition, & Federal Government of Somalia, 2019) suggests that consumption of a nutritionally sufficient diet would be cost‐prohibitive for most households in Somalia. Thus, food aid and targeted food and supplement delivery platforms are likely the most appropriate near‐term programmatic strategies in Somalia. Expanding the production, delivery and promotion of micronutrient powders (MNPs) could be used to deliver sufficient levels of iron, vitamin A and other micronutrients to children 6–59 months of age. MNPs—marketed under the name Super Fariid™ in Somalia—have been shown to be acceptable (PSI Research & Metrics, 2013) and are currently sold on a small scale in pharmacies and kiosks. Additional research examining the barriers to access and consumption should be considered to help identify approaches to expand the coverage of MNPs in Somalia, which currently cover only ~7% of children 6–59 months of age (Federal Ministry of Health et al., 2020). This research should investigate both the distribution of MNPs for purchase via market channels and for free via public food distribution programmes.

MNPs have also been developed for pregnant women; however, current WHO recommendations suggest that multiple micronutrient supplement tablets that contain iron and folic acid supplementation are recommended for pregnant women in a research context (WHO, 2020b). Among non‐pregnant Somali women, the current coverage of iron, folic acid and multivitamin syrups/tablets ranges between 9% and 11% (Federal Ministry of Health et al., 2020). This current level of consumption implies general access—albeit not widespread—to supplements, and thus, greater research examining the barriers faced by women to access supplements and the perceptions related to supplementation is warranted. Focus on expanding the coverage of supplements to adolescent girls should be prioritized in order to improve their vitamin and mineral status before and during their first pregnancy. According to Somalia's 2018–2019 Health and Demographic Survey, women give birth for the first time at (median) 20 years of age (Federal Government of Somalia, 2020).

### 4.3 Limitations

Although the SMS 2019 assessed many of the known anaemia risk factors, a large proportion of the overall anaemia remained unexplained. Kassebaum and colleagues assessed the main anaemia risk factors globally and by region (Kassebaum et al., 2014) and estimated that the primary risk factors of anaemia in adult females living in Eastern sub‐Saharan Africa were ID, schistosomiasis, malaria, hookworm infection and sickle cell disorders. Our analyses are in agreement regarding ID, but conflicting regarding malaria, which might be, as already mentioned above, due to reduced transmission during the dry season (Dery et al., 2010; Jawara et al., 2008). We did not assess schistosomiasis or hookworm infestation, but both parasites are known to be common in Somalia (Arfaa, 1975; Ilardi et al., 1987) and could account for a substantial portion of anaemia cases unexplained in our analysis. Genetic blood disorders were assessed in the children participating in the SMS 2019, but not in the women. As α‐thalassaemia was significantly associated with anaemia in children, it is likely that this could have also contributed to a small proportion of anaemia among women. Genetic blood disorders were not measured in women due to budgetary reasons, but the very low prevalence of these conditions found in children likely indicates that they are of minor importance in Somalia.

The exclusion of certain areas in Somalia due to insecurity is another limitation of our study and could potentially have biased our results. Higher levels and more severe forms of malnutrition are found in countries experiencing higher levels of conflict (Kozuki et al., 2020), and the exclusion of insecure areas in Somalia could have resulted in an underestimation in the prevalence of anaemia and ID. Though insecure areas likely had limited access to food and other key resources, it is notable that the sampling frame of the SMS included all secure areas, regardless of their accessibility. To illustrate, the SMS included Baidoa town, which, although secure, could only be accessed by plain as all other areas in the Bay region were classified as insecure.

## 5 CONCLUSION

Our analyses show that ID is the main risk factor of anaemia in both children and women in Somalia. Vitamin A deficiency is a risk factor in both groups, but does not contribute substantially to the number of cases of anaemic children and women. The risk factors of ID are not the same in children and women. Programme planners can likely reduce the prevalence of anaemia and ID by increasing the coverage of nutrition interventions that increase the intake of iron and vitamin A, such as MNPs, micronutrient supplements and vitamin A supplementation.

## CONFLICT OF INTEREST

The authors declare no competing interests. The authors alone are responsible for the views expressed in this publication, and they do not necessarily represent the decisions, policy or views of UNICEF. TNW is supported by a Senior Fellowship from Wellcome (202800/Z/16/Z). This study is published with permission from the Director of the Kenya Medical Research Institute (KEMRI).

## CONTRIBUTIONS

Conceptualization: FS, FR, NP, JPW and BAW. Formal analysis: JPW. Funding acquisition: FS and MG. Methodology: FR, JPW, NP, BAW and FS. Project administration: FS, HOY, JPW, JM, YK, FR, NP, BAW, WESD and MG. Laboratory analysis: TNW, RV and SR. Supervision: SA, YK, JM and HOY. Writing–original draft: NP and JPW.

## Supporting information


**Table S1.** Bivariate anaemia analyses children 6–59 months of age
**Table S2.** Bivariate anaemia analyses women 15–49 years
**Table S3.** Bivariate iron deficiency analyses children 6–59 months of age
**Table S4.** Bivariate iron deficiency analyses women 15–49 years

## Data Availability

The data that support the findings of this study are available from Somalia's Federal Ministry of Health (FMOH). Restrictions apply to the availability of these data, which were used with permission from Somalia's FMOH, and so are not publicly available. Data are, however, available from the corresponding author [james@groundworkhealth.org] with the permission of Somalia's FMOH.
