# Supplementary material for: Risk factors of anaemia and iron deficiency in Somali children and women: Findings from the 2019 Somalia Micronutrient Survey
Source: Matern Child Nutr. 2021 Aug 17;18(1):e13254. doi: 10.1111/mcn.13254 (PMC8710091; doi:10.1111/mcn.13254)
Supplement: Supplementary file 1 — Table S1. Bivariate anaemia analyses children 6–59 months of age Table S2. Bivariate anaemia analyses women 15–49 years Table S3. Bivariate iron deficiency analyses children 6–59 months of age Table S4. Bivariate iron deficiency analyses women 15–49 years [file MCN-18-e13254-s001.docx]

**Supplementary Table 1: Bivariate anemia analyses children 6-59 months of age**

| **Characteristic** | **Anemia ^b^** | | | |
| --- | --- | --- | --- | --- |
|  | *n* | *% ^a^* | *(95% CI) ^c^* | *P value ^d^* |
| *Age Group (in months)* |  |  |  |  |
| 6-11 | 77 | 52.7 | (43.4, 61.8) | 0.0000 |
| 12-23 | 219 | 59.7 | (52.4, 66.6) |  |
| 24-35 | 190 | 46.9 | (41.5, 52.3) |  |
| 36-47 | 162 | 42.1 | (36.2, 48.2) |  |
| 48-59 | 84 | 21.4 | (16.6, 27.1) |  |
| *Sex* |  |  |  |  |
| Male | 389 | 45.3 | (41.2, 49.5) | 0.1142 |
| Female | 343 | 41.4 | (37.2, 45.7) |  |
| *Residence* |  |  |  |  |
| Rural | 230 | 43.6 | (37.2, 50.2) | 0.1731 |
| Urban | 376 | 40.8 | (36.5, 45.3) |  |
| IDP | 126 | 52.3 | (40.3, 64.0) |  |
| *State* |  |  |  |  |
| Somaliland | 144 | 29.3 | (23.7, 35.5) | 0.0000 |
| Puntland | 117 | 43.2 | (35.8, 50.9) |  |
| Hirshabelle | 91 | 48.4 | (40.0, 56.9) |  |
| Galmudug | 43 | 29.2 | (22.5, 36.9) |  |
| South-West | 75 | 61.3 | (49.5, 71.9) |  |
| Jubaland | 63 | 53.9 | (46.9, 60.7) |  |
| Banaadir | 199 | 61.7 | (53.7, 69.0) |  |
| *Wealth quintiles* |  |  |  |  |
| Lowest | 75 | 57.0 | (47.2, 66.2) | 0.0004 |
| Second | 107 | 48.9 | (41.5, 56.4) |  |
| Middle | 186 | 41.3 | (35.2, 47.7) |  |
| Fourth | 209 | 44.7 | (39.6, 50.0) |  |
| Highest | 147 | 32.9 | (26.0, 40.5) |  |
| *Household food insecurity category* |  |  |  |  |
| Food secure | 322 | 38.8 | (34.9, 42.9) | 0.0187 |
| Mild food insecurity | 41 | 49.8 | (37.9, 61.8) |  |
| Moderate food insecurity | 84 | 52.8 | (43.2, 62.3) |  |
| Severe food insecurity | 277 | 45.4 | (40.0, 50.9) |  |
| *Household sanitation* |  |  |  |  |
| Inadequate | 377 | 44.3 | (39.7, 48.9) | 0.4579 |
| Adequate | 344 | 41.7 | (37.0, 46.6) |  |
| *Household drinking water* |  |  |  |  |
| Unsafe | 117 | 43.6 | (35.7, 51.7) | 0.9136 |
| Safe | 607 | 43.1 | (39.4, 46.9) |  |
| *Water at handwashing site* |  |  |  |  |
| No | 445 | 43.7 | (39.0, 48.6) | 0.9305 |
| Yes | 146 | 43.4 | (37.4, 49.6) |  |
| *Any soap in the house* |  |  |  |  |
| No | 276 | 47.6 | (42.2, 53.2) | 0.0277 |
| Yes | 448 | 40.2 | (36.2, 44.3) |  |
| *Sickle cell disorder* |  |  |  |  |
| No | 636 | 43.0 | (39.5, 46.6) | 0.4386 |
| Yes | 5 | 58.4 | (21.7, 87.7) |  |
| *Alpha thalassemia* |  |  |  |  |
| No | 562 | 42.0 | (38.3, 45.9) | 0.0234 |
| Yes | 60 | 57.1 | (44.5, 68.8) |  |
| *G6pd* |  |  |  |  |
| No | 617 | 42.6 | (39.0, 46.3) | 0.0525 |
| Yes | 23 | 57.7 | (42.2, 71.9) |  |
| *Diarrhea in past 2 weeks* |  |  |  |  |
| No | 641 | 43.8 | (40.4, 47.2) | 0.5872 |
| Yes | 90 | 40.9 | (33.7, 48.4) |  |
| *Cough in past 2 weeks* |  |  |  |  |
| No | 543 | 44.7 | (40.9, 48.5) | 0.3431 |
| Yes | 186 | 39.9 | (34.2, 45.9) |  |
| *Fever in past 2 weeks* |  |  |  |  |
| No | 577 | 44.4 | (40.8, 47.9) | 0.2108 |
| Yes | 153 | 39.6 | (33.3, 46.2) |  |
| *Inflammation ^f^* |  |  |  |  |
| None | 435 | 38.9 | (34.6, 43.4) | 0.0000 |
| Any inflammation | 189 | 52.1 | (47.1, 57.0) |  |
| *Inflammation ^f^* |  |  |  |  |
| None | 435 | 38.9 | (34.6, 43.4) | 0.0009 |
| Incubation (elevated CRP only) | 15 | 38.2 | (22.7, 56.6) |  |
| Early convalescence (elevated CRP and AGP) | 57 | 53.1 | (41.6, 64.3) |  |
| Late convalescence (elevated AGP only) | 117 | 54.2 | (47.5, 60.8) |  |
| *Vitamin A deficient ^g^* |  |  |  |  |
| No (RBP≥0.7 μmol/L) | 381 | 38.6 | (34.8, 42.5) | 0.0007 |
| Yes (RBP<0.7 μmol/L) | 243 | 49.3 | (43.4, 55.2) |  |
| *Iron deficient ^h^* |  |  |  |  |
| No (ferrtin≥12 μg/L) | 196 | 25.8 | (22.1, 30.0) | 0.0000 |
| Yes (ferritin<12 μg/L) | 428 | 60.7 | (55.9, 65.3) |  |
| *Stunting* |  |  |  |  |
| No | 445 | 37.8 | (33.7, 42.1) | 0.0000 |
| Yes | 141 | 56.6 | (48.8, 64.0) |  |
| *Wasting* |  |  |  |  |
| No | 520 | 40.9 | (36.7, 45.1) | 0.5643 |
| Yes | 65 | 43.4 | (35.7, 51.3) |  |
| *Weighed at birth* |  |  |  |  |
| No | 557 | 46.3 | (42.4, 50.3) | 0.0256 |
| Yes | 138 | 36.9 | (30.1, 44.3) |  |
| *Consumed formula with added iron in past 24 h* |  |  |  |  |
| No | 618 | 43.9 | (40.3, 47.6) | 0.4615 |
| Yes | 103 | 41.8 | (35.1, 48.8) |  |
| *Consumed fortified cereal in past 24 hours* |  |  |  |  |
| No | 660 | 43.5 | (39.8, 47.3) | 0.3699 |
| Yes | 60 | 44.8 | (36.5, 53.3) |  |
| *Consumed micronutrient powder in past 24 hours* |  |  |  |  |
| No | 659 | 43.1 | (39.5, 46.7) | 0.3919 |
| Yes | 61 | 49.0 | (39.3, 58.7) |  |
| *Consumed RUTF in past 24 hours* |  |  |  |  |
| No | 651 | 43.9 | (40.2, 47.6) | 0.2320 |
| Yes | 71 | 41.8 | (34.8, 49.1) |  |
| *Received vitamin A supplements in past 6 months* |  |  |  |  |
| No | 598 | 45.5 | (42.0, 49.1) | 0.0056 |
| Yes | 101 | 33.2 | (26.4, 40.8) |  |
| *Registered in a feeding program* |  |  |  |  |
| No | 618 | 42.2 | (38.5, 46.1) | 0.0444 |
| Yes | 102 | 50.6 | (43.3, 58.0) |  |
| Note: The n’s are un-weighted numerators in each subgroup; the sum of subgroups may not equal the total because of missing data.  ^a^ Percentages weighted for unequal probability of selection.  ^b^ Anemia defined as hemoglobin < 110 g/L adjusted for altitude.  ^c^ CI=confidence interval, calculated taking into account the complex sampling design.  ^d^ P-value <0.05 indicates that the proportion in at least one subgroup is statistically significantly  ^e^ Positive malaria status identified using rapid diagnostic tests results  ^f^ Incubation=CRP only; early convalescence=CRP and AGP; late convalescence=AGP only.  ^g^ Vitamin A deficiency (VAD) defined as retinol binding protein (RBP) <0.70 μmol/L; RBP concentrations adjusted for inflammation using the BRINDA approach  ^h^ Iron deficiency defined as serum ferritin < 12 µg/l, values are adjusted for inflammation according to BRINDA | | | | |

**Supplementary Table 2: Bivariate anemia analyses women 15-49 years**

| **Characteristic** | **Anemia ^b^** | | | |
| --- | --- | --- | --- | --- |
|  | *n* | *% ^a^* | *(95% CI) ^c^* | *P value ^d^* |
| *Age in years* |  |  |  |  |
| 15-19 | 58 | 34.4 | (27.3, 42.3) | 0.1746 |
| 20-24 | 63 | 38.5 | (30.2, 47.5) |  |
| 25-29 | 72 | 43.6 | (35.3, 52.2) |  |
| 30-34 | 55 | 46.2 | (35.6, 57.1) |  |
| 35-39 | 51 | 46.1 | (36.2, 56.3) |  |
| 40-44 | 26 | 38.1 | (24.9, 53.4) |  |
| 45-49 | 8 | 21.4 | (9.4, 41.8) |  |
| *Residence* |  |  |  |  |
| Rural | 93 | 41.4 | (33.7, 49.5) | 0.9041 |
| Urban | 196 | 39.4 | (34.3, 44.6) |  |
| IDP | 44 | 41.5 | (28.7, 55.5) |  |
| *State* |  |  |  |  |
| Somaliland | 64 | 27.1 | (21.9, 33.0) | 0.0000 |
| Puntland | 62 | 54.9 | (45.5, 64.0) |  |
| Hirshabelle | 29 | 59.2 | (46.5, 70.8) |  |
| Galmudug | 18 | 24.8 | (13.5, 41.2) |  |
| South-West | 23 | 50.7 | (31.1, 70.1) |  |
| Jubaland | 39 | 44.4 | (36.1, 53.1) |  |
| Banaadir | 98 | 54.3 | (47.3, 61.2) |  |
| *Woman's Education* |  |  |  |  |
| No school /informal school | 203 | 40.3 | (35.1, 45.7) | 0.1218 |
| Koranic school | 62 | 48.2 | (38.0, 58.5) |  |
| Primary school | 33 | 36.2 | (25.6, 48.2) |  |
| Secondary school | 35 | 30.7 | (21.8, 41.4) |  |
| *Wealth Quintile* |  |  |  |  |
| Lowest | 28 | 44.5 | (33.1, 56.6) | 0.0381 |
| Second | 43 | 43.4 | (33.5, 53.9) |  |
| Middle | 67 | 40.7 | (33.2, 48.6) |  |
| Fourth | 94 | 47.1 | (40.1, 54.2) |  |
| Highest | 98 | 32.6 | (27.7, 37.9) |  |
| *Household food insecurity category* |  |  |  |  |
| Food secure | 186 | 41.9 | (36.6, 47.4) | 0.6533 |
| Mild food insecurity | 9 | 29.4 | (14.9, 49.8) |  |
| Moderate food insecurity | 30 | 40.3 | (26.4, 56.0) |  |
| Severe food insecurity | 105 | 39.2 | (32.2, 46.7) |  |
| *Marital status* |  |  |  |  |
| Never married | 60 | 28.0 | (22.1, 34.8) | 0.0026 |
| Currently married | 226 | 43.4 | (38.3, 48.7) |  |
| Divorced | 44 | 49.2 | (38.1, 60.5) |  |
| Widowed | 3 | 30.6 | (9.5, 64.9) |  |
| *Pregnancy in the past 2 years* |  |  |  |  |
| No | 132 | 47.9 | (41.2, 54.7) | 0.7632 |
| Yes | 115 | 46.4 | (39.4, 53.6) |  |
| *Consumes tea or coffee* |  |  |  |  |
| No | 75 | 40.7 | (31.7, 50.4) | 0.1590 |
| Rarely | 62 | 33.1 | (25.7, 41.5) |  |
| ≥2 times per day | 196 | 43.3 | (38.1, 48.7) |  |
| *Vitamin A deficient ^g^* |  |  |  |  |
| No (RBP≥0.7 μmol/L) | 241 | 36.8 | (32.6, 41.2) | 0.0036 |
| Yes (RBP<0.7 μmol/L) | 40 | 55.6 | (42.8, 67.7) |  |
| *Iron deficient ^h^* |  |  |  |  |
| No (ferrtin≥15 μg/L) | 80 | 21.7 | (17.4, 26.8) | 0.0000 |
| Yes (ferritin<15 μg/L) | 201 | 56.0 | (50.3, 61.6) |  |
| *Folate deficient ^h^* |  |  |  |  |
| No (folate≥10 nmol/L) | 44 | 42.8 | (32.7, 53.5) | 0.2270 |
| Yes (folate<10 nmol/L) | 15 | 32.4 | (19.8, 48.1) |  |
| *Vitamin B12 deficient ^h^* |  |  |  |  |
| No (B12≥150 pmol/L) | 35 | 42.5 | (29.0, 57.2) | 0.8950 |
| Yes (B12<150 pmol/L) | 20 | 36.2 | (24.7, 49.6) |  |
| *Inflammation ^f^* |  |  |  |  |
| None | 195 | 40.4 | (35.5, 45.5) | 0.2525 |
| Any inflammation | 86 | 35.5 | (28.8, 42.9) |  |
| *Inflammation ^g^* |  |  |  |  |
| None | 195 | 40.4 | (35.5, 45.5) | 0.6911 |
| Incubation (elevated CRP only) | 40 | 36.0 | (26.6, 46.7) |  |
| Early convalescence (elevated CRP and AGP) | 32 | 33.7 | (22.6, 46.8) |  |
| Late convalescence (elevated AGP only) | 14 | 38.3 | (23.6, 55.5) |  |
| *Nutritional status* |  |  |  |  |
| Underweight | 33 | 38.7 | (28.9, 49.6) | 0.8553 |
| Normal | 162 | 40.9 | (35.1, 46.9) |  |
| Overweight | 131 | 38.8 | (32.9, 45.1) |  |
| *Minimal dietary diversity* |  |  |  |  |
| No (0-4) | 192 | 39.8 | (34.4, 45.5) | 0.7546 |
| Yes (≥5) | 141 | 40.9 | (36.1, 46.0) |  |
| *Consumed iron supplements in past 6 months* |  |  |  |  |
| No | 295 | 40.3 | (36.0, 44.7) | 0.9684 |
| Yes | 37 | 40.1 | (29.7, 51.6) |  |
| *Consumed folic acid supplements in past 6 months* |  |  |  |  |
| No | 297 | 39.9 | (35.7, 44.2) | 0.3833 |
| Yes | 36 | 45.1 | (34.0, 56.8) |  |
| *Consumed multi-nutrient supplements in past 6 months* |  |  |  |  |
| No | 288 | 39.8 | (35.2, 44.6) | 0.7587 |
| Yes | 44 | 44.0 | (33.2, 55.3) |  |
| *Household sanitation* |  |  |  |  |
| Inadequate | 164 | 43.4 | (36.8, 50.4) | 0.1249 |
| Adequate | 165 | 37.2 | (32.8, 41.8) |  |
| *Household drinking water* |  |  |  |  |
| Unsafe | 48 | 38.1 | (27.8, 49.6) | 0.2960 |
| Safe | 282 | 40.7 | (36.3, 45.3) |  |
| *Water at handwashing site* |  |  |  |  |
| No | 221 | 40.9 | (36.1, 45.9) | 0.0232 |
| Yes | 62 | 41.6 | (31.9, 51.9) |  |
| *Any soap in the house* |  |  |  |  |
| No | 111 | 43.2 | (34.9, 52.0) | 0.3313 |
| Yes | 219 | 38.5 | (34.4, 42.8) |  |
| *Consumed organ meat in past 24 hours* |  |  |  |  |
| No | 195 | 41.3 | (35.5, 47.4) | 0.6010 |
| Yes | 133 | 39.1 | (32.9, 45.7) |  |
| *Consumed any meat in past 24 hours* |  |  |  |  |
| No | 163 | 37.5 | (32.3, 43.2) | 0.1783 |
| Yes | 165 | 44.5 | (37.7, 51.6) |  |
| *Consumed fish or sea food in past 24 hours* |  |  |  |  |
| No | 234 | 40.0 | (34.9, 45.3) | 0.5300 |
| Yes | 96 | 41.9 | (35.4, 48.7) |  |
| Note: The n’s are un-weighted numerators in each subgroup; the sum of subgroups may not equal the total because of missing data.  ^a^ Percentages weighted for unequal probability of selection.  ^b^ Anemia defined as hemoglobin < 110 g/L adjusted for altitude.  ^c^ CI=confidence interval, calculated taking into account the complex sampling design.  ^d^ P-value <0.05 indicates that the proportion in at least one subgroup is statistically significantly  ^e^ Positive malaria status identified using rapid diagnostic tests results  ^f^ Incubation=CRP only; early convalescence=CRP and AGP; late convalescence=AGP only.  ^g^ Vitamin A deficiency (VAD) defined as retinol binding protein (RBP) <0.70 μmol/L; RBP concentrations adjusted for inflammation using the BRINDA approach  ^h^ Iron deficiency defined as serum ferritin < 12 µg/l, values are adjusted for inflammation according to BRINDA | | | | |

**Supplementary Table 3: Bivariate iron deficiency analyses children 6-59 months of age**

| **Characteristic** | **Iron deficiency ^b^** | | | |
| --- | --- | --- | --- | --- |
|  | **n** | **% ^a^** | **(95% CI) ^c^** | **P value ^d^** |
| *Age Group (in months)* |  |  |  |  |
| 6-11 | 57 | 49.8 | (39.9, 59.7) | 0.0000 |
| 12-23 | 196 | 62.0 | (56.0, 67.6) |  |
| 24-35 | 195 | 54.5 | (47.5, 61.3) |  |
| 36-47 | 158 | 43.4 | (37.4, 49.6) |  |
| 48-59 | 99 | 29.1 | (23.1, 36.0) |  |
| *Sex* |  |  |  |  |
| Male | 367 | 49.8 | (44.9, 54.6) | 0.0801 |
| Female | 338 | 44.6 | (40.0, 49.3) |  |
| *Residence* |  |  |  |  |
| Rural | 240 | 48.2 | (41.5, 55.0) | 0.8545 |
| Urban | 362 | 46.2 | (41.1, 51.4) |  |
| IDP | 103 | 48.6 | (39.1, 58.2) |  |
| *State* |  |  |  |  |
| Somaliland | 214 | 44.9 | (39.3, 50.5) | 0.0087 |
| Puntland | 110 | 46.1 | (37.3, 55.1) |  |
| Hirshabelle | 92 | 55.1 | (43.9, 65.8) |  |
| Galmudug | 36 | 23.3 | (12.1, 40.2) |  |
| South-West | 44 | 53.0 | (36.2, 69.1) |  |
| Jubaland | 47 | 46.3 | (35.1, 57.9) |  |
| Banaadir | 162 | 59.3 | (52.9, 65.5) |  |
| *Wealth Quintile* |  |  |  |  |
| Lowest | 53 | 48.5 | (39.2, 58.0) | 0.3673 |
| Second | 96 | 48.1 | (38.2, 58.1) |  |
| Middle | 181 | 45.0 | (38.5, 51.6) |  |
| Fourth | 207 | 52.3 | (45.9, 58.6) |  |
| Highest | 162 | 42.9 | (36.6, 49.6) |  |
| *Household food insecurity category* |  |  |  |  |
| Food secure | 322 | 45.1 | (39.8, 50.5) | 0.1920 |
| Mild food insecurity | 47 | 59.4 | (48.6, 69.4) |  |
| Moderate food insecurity | 76 | 49.0 | (39.7, 58.4) |  |
| Severe food insecurity | 254 | 47.3 | (41.8, 53.0) |  |
| *Household sanitation* |  |  |  |  |
| Inadequate | 357 | 47.9 | (43.2, 52.7) | 0.3923 |
| Adequate | 338 | 45.9 | (40.8, 51.0) |  |
| *Household drinking water* |  |  |  |  |
| Unsafe | 123 | 47.7 | (39.3, 56.3) | 0.8572 |
| Safe | 576 | 46.9 | (42.8, 51.0) |  |
| *Water at handwashing site* |  |  |  |  |
| No | 419 | 48.3 | (43.9, 52.7) | 0.8186 |
| Yes | 155 | 47.5 | (41.5, 53.5) |  |
| *Any soap in the house* |  |  |  |  |
| No | 230 | 45.4 | (38.7, 52.2) | 0.4972 |
| Yes | 469 | 48.1 | (43.7, 52.5) |  |
| *Sickle cell disorder* |  |  |  |  |
| No | 670 | 47.9 | (43.9, 51.9) | 0.9116 |
| Yes | 5 | 45.9 | (16.5, 78.4) |  |
| *Alpha thalassemia* |  |  |  |  |
| No | 607 | 48.7 | (44.6, 52.8) | 0.1094 |
| Yes | 44 | 38.6 | (27.5, 51.1) |  |
| *G6pd* |  |  |  |  |
| No | 655 | 47.9 | (43.9, 51.9) | 0.6965 |
| Yes | 18 | 43.6 | (24.5, 64.9) |  |
| *Diarrhea in past 2 weeks* |  |  |  |  |
| No | 616 | 47.3 | (43.4, 51.2) | 0.7828 |
| Yes | 88 | 46.2 | (38.3, 54.2) |  |
| *Cough in past 2 weeks* |  |  |  |  |
| No | 497 | 46.1 | (41.8, 50.5) | 0.1647 |
| Yes | 207 | 50.5 | (44.9, 56.2) |  |
| *Fever in past 2 weeks* |  |  |  |  |
| No | 546 | 47.4 | (43.1, 51.7) | 0.5253 |
| Yes | 158 | 46.4 | (41.2, 51.8) |  |
| *Inflammation ^f^* |  |  |  |  |
| None | 532 | 48.1 | (43.8, 52.5) | 0.3041 |
| Any inflammation | 173 | 44.5 | (38.7, 50.5) |  |
| *Inflammation ^f^* |  |  |  |  |
| None | 532 | 48.1 | (43.8, 52.5) | 0.0548 |
| Incubation (elevated CRP only) | 16 | 33.0 | (19.5, 50.1) |  |
| Early convalescence (elevated CRP and AGP) | 41 | 36.1 | (26.4, 47.1) |  |
| Late convalescence (elevated AGP only) | 116 | 50.8 | (42.8, 58.7) |  |
| *Vitamin A deficient ^g^* |  |  |  |  |
| No (RBP≥0.7 μmol/L) | 418 | 42.5 | (38.2, 47.0) | 0.0000 |
| Yes (RBP<0.7 μmol/L) | 287 | 56.2 | (51.0, 61.2) |  |
| *Stunting* |  |  |  |  |
| No | 450 | 44.8 | (40.4, 49.2) | 0.0000 |
| Yes | 135 | 66.4 | (59.6, 72.6) |  |
| *Wasting* |  |  |  |  |
| No | 533 | 49.4 | (45.6, 53.3) | 0.0881 |
| Yes | 53 | 40.6 | (31.2, 50.9) |  |
| *Weighed at birth* |  |  |  |  |
| No | 500 | 46.0 | (41.6, 50.5) | 0.4715 |
| Yes | 158 | 48.9 | (42.1, 55.8) |  |
| *Consumed formula with added iron in past 24 h* |  |  |  |  |
| No | 585 | 47.4 | (43.2, 51.7) | 0.7899 |
| Yes | 106 | 46.8 | (39.8, 53.9) |  |
| *Consumed fortified cereal in past 24 hours* |  |  |  |  |
| No | 640 | 48.1 | (44.2, 52.1) | 0.0340 |
| Yes | 54 | 42.0 | (33.9, 50.6) |  |
| *Consumed micronutrient powder in past 24 hours* |  |  |  |  |
| No | 637 | 47.0 | (43.0, 51.0) | 0.6999 |
| Yes | 56 | 50.9 | (40.9, 60.8) |  |
| *Consumed RUTF in past 24 hours* |  |  |  |  |
| No | 629 | 48.0 | (44.0, 52.1) | 0.1497 |
| Yes | 66 | 43.3 | (34.5, 52.4) |  |
| *Received vitamin supplements in past 6 months* |  |  |  |  |
| No | 553 | 47.3 | (43.1, 51.5) | 0.8813 |
| Yes | 120 | 46.2 | (39.0, 53.6) |  |
| *Registered in a feeding program* |  |  |  |  |
| No | 606 | 47.8 | (43.8, 51.9) | 0.1850 |
| Yes | 88 | 45.5 | (39.1, 52.1) |  |
| Note: The n’s are un-weighted numerators in each subgroup; the sum of subgroups may not equal the total because of missing data.  ^a^ Percentages weighted for unequal probability of selection.  ^b^ Anemia defined as hemoglobin < 110 g/L adjusted for altitude.  ^c^ CI=confidence interval, calculated taking into account the complex sampling design.  ^d^ P-value <0.05 indicates that the proportion in at least one subgroup is statistically significantly  ^e^ Positive malaria status identified using rapid diagnostic tests results  ^f^ Incubation=CRP only; early convalescence=CRP and AGP; late convalescence=AGP only.  ^g^ Vitamin A deficiency (VAD) defined as retinol binding protein (RBP) <0.70 μmol/L; RBP concentrations adjusted for inflammation using the BRINDA approach  ^h^ Iron deficiency defined as serum ferritin < 12 µg/l, values are adjusted for inflammation according to BRINDA | | | | |

**Supplementary Table 4: Bivariate iron deficiency analyses women 15-49 years**

| **Characteristic** | **Iron deficiency ^b^** | | | |
| --- | --- | --- | --- | --- |
|  | **n** | **% ^a^** | **(95% CI) ^c^** | **P value ^d^** |
| *Age in years* |  |  |  |  |
| 15-19 | 65 | 44.8 | (35.2, 54.8) | 0.5572 |
| 20-24 | 73 | 52.2 | (44.0, 60.3) |  |
| 25-29 | 69 | 49.1 | (40.6, 57.7) |  |
| 30-34 | 55 | 57.4 | (46.0, 68.2) |  |
| 35-39 | 48 | 49.9 | (39.1, 60.7) |  |
| 40-44 | 26 | 50.0 | (35.6, 64.4) |  |
| 45-49 | 9 | 37.4 | (21.7, 56.3) |  |
| *Residence* |  |  |  |  |
| Rural | 89 | 51.1 | (43.1, 58.9) | 0.7885 |
| Urban | 210 | 49.9 | (44.6, 55.3) |  |
| IDP | 46 | 46.0 | (33.2, 59.4) |  |
| *State* |  |  |  |  |
| Somaliland | 112 | 49.5 | (42.0, 57.1) | 0.0005 |
| Puntland | 64 | 65.2 | (54.6, 74.5) |  |
| Hirshabelle | 15 | 48.4 | (32.5, 64.6) |  |
| Galmudug | 10 | 16.4 | ( 8.4, 29.7) |  |
| South-West | 23 | 52.1 | (40.0, 63.9) |  |
| Jubaland | 40 | 51.6 | (42.1, 61.0) |  |
| Banaadir | 81 | 50.5 | (42.4, 58.6) |  |
| *Woman's Education* |  |  |  |  |
| No school /informal school | 217 | 52.9 | (47.4, 58.3) | 0.1092 |
| Koranic school | 55 | 49.3 | (39.8, 58.9) |  |
| Primary school | 35 | 44.0 | (32.7, 56.0) |  |
| Secondary school | 38 | 37.6 | (26.4, 50.3) |  |
| *Wealth Quintile* |  |  |  |  |
| Lowest | 37 | 61.3 | (47.0, 73.8) | 0.0608 |
| Second | 34 | 38.0 | (26.5, 51.1) |  |
| Middle | 67 | 52.7 | (41.0, 64.1) |  |
| Fourth | 91 | 55.6 | (47.6, 63.2) |  |
| Highest | 111 | 45.7 | (38.2, 53.4) |  |
| *Household food insecurity category* |  |  |  |  |
| Food secure | 184 | 51.0 | (44.7, 57.3) | 0.5856 |
| Mild food insecurity | 16 | 61.4 | (34.1, 83.0) |  |
| Moderate food insecurity | 32 | 47.2 | (32.9, 62.0) |  |
| Severe food insecurity | 108 | 46.7 | (40.2, 53.3) |  |
| *Marital status* |  |  |  |  |
| Never married | 80 | 44.4 | (36.5, 52.7) | 0.1129 |
| Currently married | 229 | 52.8 | (47.8, 57.7) |  |
| Divorced | 32 | 46.8 | (35.2, 58.7) |  |
| Widowed | 4 | 26.1 | ( 7.9, 59.1) |  |
| *Pregnancy in the past 2 years* |  |  |  |  |
| No | 105 | 45.9 | (39.3, 52.7) | 0.0016 |
| Yes | 130 | 61.2 | (54.0, 68.0) |  |
| *Consumes tea or coffee* |  |  |  |  |
| No | 83 | 49.0 | (40.5, 57.6) | 0.6791 |
| Rarely | 81 | 53.4 | (42.5, 63.9) |  |
| ≥2 times per day | 181 | 48.3 | (42.8, 53.9) |  |
| *Vitamin A deficient ^g^* |  |  |  |  |
| No (RBP≥0.7 μmol/L) | 300 | 48.7 | (43.9, 53.4) | 0.1260 |
| Yes (RBP<0.7 μmol/L) | 45 | 58.6 | (47.1, 69.2) |  |
| *Folate deficient ^h^* |  |  |  |  |
| No (folate≥10 nmol/L) | 50 | 55.6 | (45.0, 65.6) | 0.9780 |
| Yes (folate<10 nmol/L) | 24 | 55.8 | (40.6, 70.0) |  |
| *Vitamin B12 deficient ^h^* |  |  |  |  |
| No (B12≥150 pmol/L) | 46 | 59.0 | (47.6, 69.5) | 0,2830 |
| Yes (B12<150 pmol/L) | 21 | 48.8 | (34.1, 63.8) |  |
| *Inflammation ^f^* |  |  |  |  |
| None | 237 | 51.2 | (46.1, 56.3) | 0.3098 |
| Any inflammation | 108 | 46.8 | (39.6, 54.0) |  |
| *Inflammation ^g^* |  |  |  |  |
| None | 237 | 51.2 | (46.1, 56.3) | 0.0230 |
| Incubation (elevated CRP only) | 50 | 45.7 | (34.7, 57.1) |  |
| Early convalescence (elevated CRP and AGP) | 34 | 37.5 | (26.8, 49.5) |  |
| Late convalescence (elevated AGP only) | 24 | 70.2 | (50.8, 84.3) |  |
| *Nutritional status* |  |  |  |  |
| Underweight | 43 | 58.4 | (44.4, 71.2) | 0.3439 |
| Normal | 155 | 48.5 | (42.6, 54.3) |  |
| Overweight | 144 | 48.9 | (43.0, 54.9) |  |
| *Minimal dietary diversity* |  |  |  |  |
| No (0-4) | 188 | 47.3 | (41.9, 52.8) | 0.1162 |
| Yes (≥5) | 157 | 53.7 | (47.4, 59.8) |  |
| *Consumed iron supplements in past 6 months* |  |  |  |  |
| No | 301 | 49.1 | (44.7, 53.5) | 0.3530 |
| Yes | 42 | 53.4 | (40.5, 65.8) |  |
| *Consumed folic acid supplements in past 6 months* |  |  |  |  |
| No | 310 | 49.5 | (45.1, 54.0) | 0.4781 |
| Yes | 34 | 50.2 | (39.1, 61.2) |  |
| *Consumed multi-nutrient supplements in past 6 months* |  |  |  |  |
| No | 302 | 49.5 | (45.0, 54.0) | 0.1234 |
| Yes | 39 | 48.8 | (38.2, 59.5) |  |
| *Household sanitation* |  |  |  |  |
| Inadequate | 156 | 47.2 | (40.9, 53.5) | 0.2540 |
| Adequate | 183 | 52.3 | (46.4, 58.1) |  |
| *Household drinking water* |  |  |  |  |
| Unsafe | 53 | 50.2 | (40.1, 60.3) | 0.8961 |
| Safe | 287 | 49.5 | (45.1, 54.0) |  |
| *Water at handwashing site* |  |  |  |  |
| No | 211 | 46.8 | (41.8, 51.8) | 0.2345 |
| Yes | 68 | 52.5 | (44.0, 60.9) |  |
| *Any soap in the house* |  |  |  |  |
| No | 93 | 41.1 | (35.6, 46.9) |  |
| Yes | 247 | 54.9 | (49.4, 60.3) |  |
| *Consumed organ meat in past 24 hours* |  |  |  |  |
| No | 185 | 46.0 | (41.1, 50.9) | 0.0090 |
| Yes | 153 | 55.7 | (49.1, 62.1) |  |
| *Consumed any meat in past 24 hours* |  |  |  |  |
| No | 179 | 48.0 | (42.1, 54.0) | 0.4786 |
| Yes | 159 | 51.8 | (45.4, 58.1) |  |
| *Consumed fish or sea food in past 24 hours* |  |  |  |  |
| No | 243 | 49.9 | (44.9, 54.8) | 0.9850 |
| Yes | 98 | 49.2 | (40.2, 58.4) |  |
| Note: The n’s are un-weighted numerators in each subgroup; the sum of subgroups may not equal the total because of missing data.  ^a^ Percentages weighted for unequal probability of selection.  ^b^ Anemia defined as hemoglobin < 110 g/L adjusted for altitude.  ^c^ CI=confidence interval, calculated taking into account the complex sampling design.  ^d^ P-value <0.05 indicates that the proportion in at least one subgroup is statistically significantly  ^e^ Positive malaria status identified using rapid diagnostic tests results  ^f^ Incubation=CRP only; early convalescence=CRP and AGP; late convalescence=AGP only.  ^g^ Vitamin A deficiency (VAD) defined as retinol binding protein (RBP) <0.70 μmol/L; RBP concentrations adjusted for inflammation using the BRINDA approach  ^h^ Iron deficiency defined as serum ferritin < 12 µg/l, values are adjusted for inflammation according to BRINDA | | | | |
